# Prevalence and associations for use of a traditional medicine provider in the SAMINOR 1 Survey: a population-based study on Health and Living Conditions in Regions with Sami and Norwegian Populations

**DOI:** 10.1186/s12906-017-2037-0

**Published:** 2017-12-12

**Authors:** Agnete Egilsdatter Kristoffersen, Trine Stub, Marita Melhus, Ann Ragnhild Broderstad

**Affiliations:** 1 National Research Center in Complementary and Alternative Medicine (NAFKAM), Department of Community Medicine, Faculty of Health Science, UiT The Arctic University of Norway, Tromsø, Norway; 2 Centre for Sami Health Research, Department of Community Medicine, Faculty of Health Science, UiT The Arctic University of Norway, Tromsø, Norway; 3 Department of Medicine, University Hospital of North Norway, Harstad, Norway

**Keywords:** Traditional medicine, Traditional healing, Spiritual healing, Sami, Norway, Religious healing

## Abstract

**Background:**

In Northern Norway, traditional medicine (TM) is shaped by both Christianity and traditional Sami nature worship. The healing rituals may include prayer and the use of tools such as moss, water, stones, wool and soil. Examples of TM modalities offered is cupping, blood-stemming, laying on of hands, healing prayers, and rituals. The purpose of this study was to investigate the prevalence of the use of TM in areas with predominantly Sami and Norwegian populations, and the influence of ethnicity, geography, gender, age, education, household income, religiosity and self-reported health on such use.

**Methods:**

The study is based on data collected in the first SAMINOR Survey (SAMINOR 1) conducted in 2003/2004, including three self-administered questionnaires, clinical measures, and blood analyses. Data was collected in 24 municipalities in Norway known to have a substantial population of Sami. All residents aged 30 and 36–78/79 years in the predefined regions were invited regardless of ethnic background (*N* = 27,987). Of these, 16,865 (60.3%) accepted to participate and gave their consent to medical research.

**Results:**

Of the 16,544 people responding to the question about TM use, 2276 (13.8%) reported to have used TM once or more during their lifetime. The most outstanding characteristic of the TM users was the affiliation to the Laestadian church, where 34.3% (*n* = 273) reported such use, followed by an inner Finnmark residence (31.1%, *n* = 481) and a Sami ethnicity (25.7%, *n* = 1014). Women were slightly more likely to use TM compared to men (15.9% and 11.5% accordingly, *p* < 0.001), and the TM users were slightly younger than the non-TM users (mean age 52.3 versus 54.3 years, *p* < 0.001). The TM users also had lower income (*p* < 0.001) than the non-TM users. We found no significant differences between the TM users and the non-TM users concerning years of education, and whether the participants were living with a spouse/partner or not.

**Conclusion:**

Further studies are necessary to examine the development of TM use in Norway over time, and use in areas with mainly Norwegian inhabitants. There is also a lack of studies quantifying TM use among Sami people in Sweden, Finland and Russia.

## Background

**Fig. 1:**
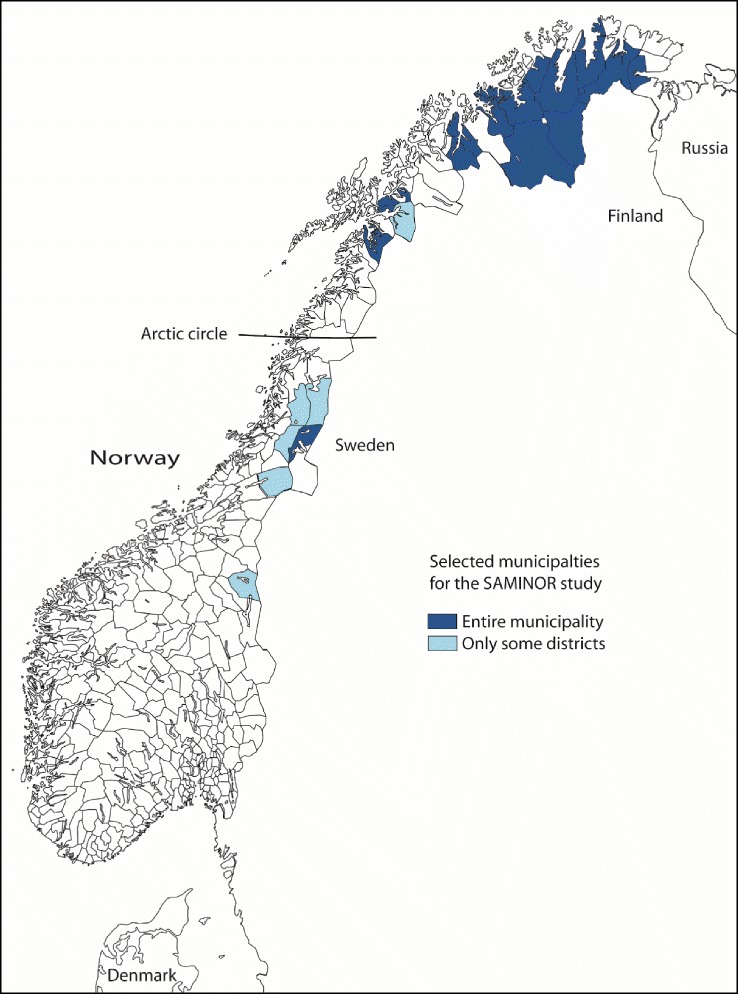
Map of the Sami settlements in Norway and the municipalities included in the four geographical regions. Republished with permission from Center for Sami Health Research

There have always been people of different ethnic backgrounds in Northern Norway. They speak different languages and belong to different cultures, such as Sami, Kven (Finnish descent) and Norwegians [1]. The Sami is a group of people with Finno-Ugric origin, settled in the northern part of Norway, Sweden, Finland and Russia and the only indigenous people in Scandinavia. Traditionally the Sami lived as farmers and fishermen or with a semi-nomadic life as reindeer herders [2, 3]. The largest Sami population is found in Norway where they have their own language, cultural history, rights [3, 4] and a Sami parliament [5]. No reliable or updated demographic record of the Sami exists. The accurate number of Sami people living in Norway today is not clear as ethnicity is not registered in public registers [6]. Many inhabitants of Northern Norway have in addition a mixture of Sami, Kven and Norwegian ancestors. Estimates, however, vary between 40,000 and 80,000 in accordance with the criteria used (heritage, mother tongue and sense of belonging to the Sami etc.). It is a great deal of diversity regarding Sami affiliation across the geographical regions within the Sami population. The traditional Sami settlements are demonstrated in Fig. 1, although the Sami people today live all over Norway.

Complementary and Alternative Medicine (CAM) is defined as a treatment modality that is used alongside conventional treatments, but not considered standard medical treatments [7]. The most commonly used CAM modalities in Norway are massage, acupuncture, naprapathy, reflexology, osteopathy, cupping and healing [8]. Among the CAM modalities offered in Norway is *traditional* healing, also categorized as traditional medicine (TM). TM draws on a long history and is understood as “the sum total of the knowledge, skills, and practices based on the theories, beliefs, and experiences indigenous to different cultures […], used in the maintenance of health as well as in the prevention, diagnosis, improvement or treatment of physical and mental illness” [9]. The term traditional medicine is in some countries used interchangeably with complementary and alternative medicine [10]. In Norway, however, TM is considered a treatment modality under the CAM umbrella.

The TM used in Northern Norway is shaped by both Christianity and traditional Sami nature worship [11]. The healing rituals may include prayer and the use of tools such as moss, water, stones, wool and soil [12]. Examples of TM modalities offered in Northern Norway is cupping, blood-stemming, laying on of hands, healing prayers (called *reading*), and rituals [13]. The healing gifts are passed from healers to selected people who show signs of healing abilities or a calling for such work - often a younger member within the healer’s close family [14].

The traditional healers are mostly non-professional and non-commercial therapists [13, 15]. Treatment is often available within the family and mostly free of charge. In 1975 Efskind and Johansen found that 34% (*n* = 41) of the participants in a survey conducted in a small town in Northern Norway reported to have used TM sometime in the past [16]. In 2005, Sørlie et al. found that 50% (*n* = 34) of the patients following psychiatric hospital treatment reported previous visits to TM providers. They found this more often among Sami patients (64.5%, *n* = 20) than among Norwegian patients (37.8%, *n* = 14) [17]. In 2006 Bakken et al. found that modern or traditional healing was used by 16% (*n* = 2675) of the participants in the first population-based study on Health and Living Conditions in Regions with Sami and Norwegian Populations (the SAMINOR 1 Survey). She also found that participants with a Sami background were more frequent users of healing than the non-Sami participants [18].

Knowledge about the use of TM is important to secure a patient centered health care. While several qualitative studies gathered important knowledge about how TM is practiced, studies quantifying the use in different groups are still limited.

Thus, the purpose of this study was to investigate the prevalence of the use of TM in areas with predominantly Sami and Norwegian populations, and the influence of ethnicity, geography, gender, age, education, household income, religiosity and self-reported health on such use.

## Methods

### Participants

#### The SAMINOR Survey

The SAMINOR Survey was designed to provide more information about health and living conditions in areas with mixed Sami and Norwegian settlements in Norway. The first survey (SAMINOR 1) was carried out between January 2003 and April 2004 and included 16,865 participants. The second survey (SAMINOR 2) was conducted in two steps. The SAMINOR 2 *Questionnaire* Survey was carried out between January 2012 and October 2012 and included 11,600 participants. The SAMINOR 2 *Clinical* Survey was carried out between September 2012 and June 2014 and included 6004 participants.

This study was based on data collected in the SAMINOR 1 Survey [2] as the SAMINOR 2 Survey did not include questions regarding use of TM. SAMINOR 1 was conducted by the Centre for Sami Health Research in collaboration with the Norwegian Institute of Public Health. The chosen areas were based on areas where more than 5–10% of the population reported themselves to be Sami [2] in the 1970 census, as described in the report *The Lappish population in Northern Norway* [19]. In addition, historical and local knowledge were taken into account.

The SAMINOR 1 Survey included three self-administered questionnaires, clinical measures, and blood analyses. Data was collected in 24 municipalities (Fig. 1), known to have a substantial population of Sami, though only selected districts were included in some of the municipalities. Except for the municipality of Alta, all included municipalities were small (<3000 inhabitants) and rural. All residents aged 30 and 36–78/79 years were invited, regardless of ethnic background (*n* = 27,987). Of these, 16,865 (60.3%) accepted to participate and gave their consent to medical research. This study was based on data retrieved from the questionnaires. The initial questionnaire (Q1), including the questions about TM use, ethnicity and income was completed by 16,544 (response rate 59.1%). Of these 15,730 completed the second questionnaire (Q2) including the health related questions while the third questionnaire (Q3) including church affiliation was completed by 13,547. The response rate was higher for women and in the older (36–78/9) age group. In the first four municipalities, the first questionnaire (Q1) followed the invitation to participate in the study. A second questionnaire (Q2) was sent out alongside the letter confirming the time and place for the clinical examination. In the remaining municipalities, the first and second questionnaires were combined and sent together with an invitation to participate in a clinical examination. After the clinical examination, the participants were asked to answer a third questionnaire (Q3) and return this by post.

#### Ethnicity

Ethnicity was determined by eleven questions regarding home language, ethnic background and self-perceived ethnicity/identity: *What language(s) do/did you, your parents and your grandparents use at home?* The questions were to be answered separately for each relative. The response categories were “*Norwegian*”, “*Sami*”, “*Kven*” (Finnish immigrants), or “*Other*”. Providing the same response options, we asked: *What is your, your father’s and your mother’s ethnic background?* The respondents also reported whether they considered themselves to be *Norwegian*, *Sami*, *Kven* or *other*. On all these questions, multiple answers were allowed. Based on these questions, participants were categorized into three groups: Sami, Sami affiliation or Non-Sami.

**Sami** was defined by customization (counts himself as Sami or reported having Sami ethnic background) and language criterion (Sami language in at least one of their grandparents, parents or themselves).

**Sami affiliation** was defined by crossing Sami for at least one question, without meeting the full criteria above.

**Non-Sami**: Those who were not included in the two above mentioned categories including Norwegians, Kvens and others.

### Geographical regions

**Fig. 2:**
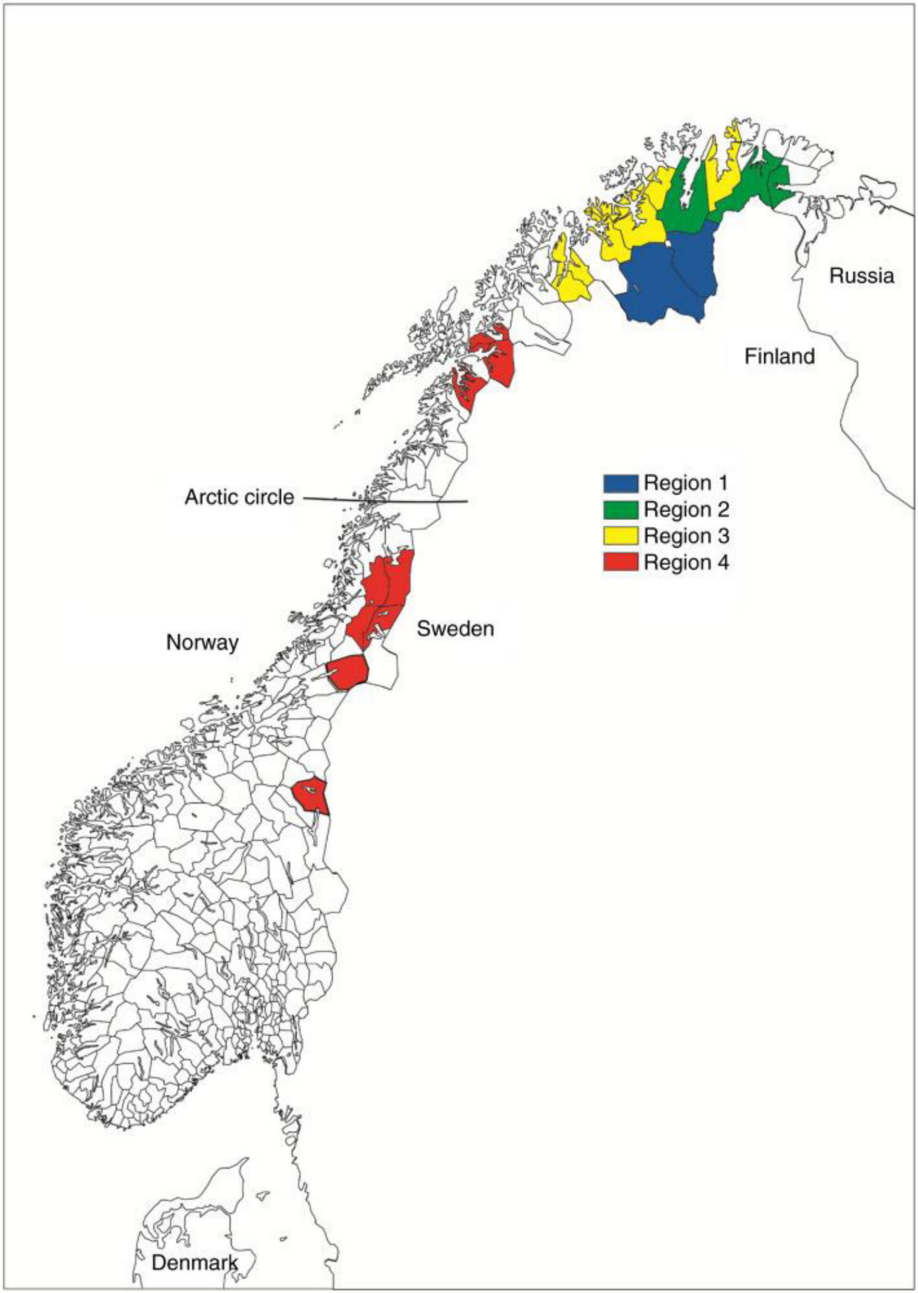
Selected municipalities for the SAMINOR 1 survey. Republished with permission from Center for Sami Health Research

In line with Naseribafrouei et al. [20], we defined four geographical regions (Fig. 2):

- **Region 1:** The inland of Finnmark county, including Karasjok and Kautokeino.
- **Region 2:** The inland and coastal areas of Finnmark county, including Porsanger, Tana and Nesseby.
- **Region 3:** The coastal areas of Finnmark and the northern part of Troms county, including Lyngen, Storfjord, Kåfjord, Kvænangen, Alta, Loppa, Kvalsund and Lebesby.
- **Region 4:** Marka, Lule and South Sami areas in Southern Troms, Nordland, Nord- and Sør-Trøndelag counties, including Lavangen, Evenes, Skånland, Tysfjord, and Røyrvik. In addition, some selected school districts were included: Vassdalen in Narvik municipality, Hattfjelldal in Hattfjelldal municipality, Trones and Furuly in Namsskogan, Majavatn in Grane, Vinje in Snåsa, and Brekken in Røros.

### Measures

#### Use of traditional medicine

In this study, a user of TM was measured by visits to a TM provider (*traditional healer*). The TM provider asked for in the questionnaire (*guvllar, reader, blower, hands on healer*) are known to base their practice on religion and religious rituals, such as prayers and the laying on of hands. Many have special abilities such as warm hands and clairvoyance [21]. In addition, many use herbs and rituals as a part of their service [12].

In the questionnaire the participants were asked “*If you have ever used alternative providers, which have you used*?” The responses were *traditional healer (guvllar, reader, blower, hands on healer*); *modern healer; acupuncturist; reflexologist, homeopath, kinesiologist* (etc). All participants who checked for *traditional healer (guvllar, reader, blower, hands on healer)* were considered users of TM. There was not provided any definition of a *traditional healer* apart from the examples given in the parenthesis (*guvllar, reader, blower, hands on healer*).

#### Self-reported health and health complaints

The general health condition was measured as a response to the question: *How is your current health?* where we merged the original four responses: *Poor*, *not good*, *good* and *excellent* into *poor* and *good* health.

The report of spesific health complaints were mainly reported as a *yes*-response to the question: *Do you have or have you had….* followed by the listed complaints. Exceptions were daily cough, which was a yes-response to the question *Do you cough more or less daily for some periods of the year?;* insomnia, which was a yes-response to *Do you from time to time suffer from sleeplessness/insomnia?* and sad/depressed, which was a yes-response to *Does it happen that you for longer periods (>14 days) feel sad and depressed?*

The questions *During the last 14 days, have you felt unable to cope with your difficulties?*and *Do you sometimes feel lonely?* have alle avilable response categories presented in Table 2. Fracture was a yes-response to either fracture in wrist/underarm or fracture in the femoral/neck.

#### Socio-demographic variables

We defined three education response categories from the original continuous variable referring to the number of years of education: Primary education (0–9 years), secondary education (10–12 years) and college/university education (13 years or more). The income variable referred to the household’s total gross income in the previous year. Six original response categories were merged into low income (< NOK 300,000 (€ 32,000)), middle income (NOK 300,000–600,000 (€ 32,000–63,000)) and high income (> NOK 600,000 (€ 63,000).

#### Church affiliation

The church affiliation was mapped through the following question: *Do you have an affiliation to any of the following churches/religious church communities?* With the options: *Member of The Church of Norway*; *The Laestadian church*; *Other church community*; *Not a member of any church*.

The Church of Norway is a Lutheran denomination of Protestant Christianity that serves as the people’s church of Norway. It is by far the largest church in Norway, and until the nineteenth century membership was mandatory for everyone [22]. Today the church of Norway has 3,758,070 members constituting 71.5% of the total population [23]. Laestadianism is a conservative Lutheran revival movement started in Lapland in the middle of the nineteenth century, named after the Swedish state church administrator and temperance movement leader Lars Levi Laestadius. It has members mainly in Finland, North America, Norway, Russia and Sweden [24, 25]. The number of Laestadians worldwide is estimated at between 144,000 and 219,000 [24] of which 50,000 [26] are estimated to live in Norway.

### Statistical analysis

Between-group differences were analyzed using the Pearson’s chi-square tests for binary data analyzing one variable at the time and one-way ANOVA test for continuous data in SPSS for Windows (version 24.0, SPSS, Inc., Chicago, IL). The significance level was defined as *p* < 0.05 without *p*-value adjustment for multiple comparisons.

## Results

### Basic characteristics of the participants

As shown in Table 1, the mean age of the participants was 54 years with a range of 30–79 years. There were more women (51.8%, *n* = 8571) than men (48.2% *n* = 7973). Most of the participants had low to middle income (88.7%, *n* = 13,241) and lived by the coast (77.8%, *n* = 12,865), mainly in region 3 (coastal areas in Finnmark and the northern part of Troms, 53.2%, *n* = 8809), classified as Non-Sami (64.6%, *n* = 10,649) and were members of Church of Norway (83.7%, *n* = 11,121) (Table 1, left column). The Non-Sami group consisted mainly of Norwegians (93.6%, *n* = 9969). The rest considered themselves either as Kvens (1.8%, *n* = 196) or “other” (4.5%, *n* = 335) (Table 1).

**Table 1:** Basic characteristics of the total sample and among users and non-users of TM

	Total sample		TM users		No TM users		*p*-value*
	%	n**	%	n	%	n	
Age							
Mean	54.05	16,544	52.3	2276	54.3	14,268	<0.001
Range	(30–79)	16,544	30–78	2276	30–79	14,268	
Gender							<0.001
Men	48.2	7973	11.5	914	88.5	7196	
Women	51.8	8571	15.9	1362	84.1	7393	
Living with a spouse/partner							0.089
Yes	76.6	10,176	13.4	1366	86.6	8810	
No	23.4	3105	14.6	454	85.4	2651	
Household income							<0.001
Low (< NOK 300′/ € 32′)	38.9	5808	15.6	907	84.4	4901	
Middle (NOK 300′-600′/€ 32′-63′)	49.8	7433	13.6	1008	86.4	6425	
High (>NOK 600′/€ 63′)	11.3	1685	11.8	199	88.2	1486	
Years of Education							0.250
Primary (0–9 years)	37.0	5465	14.3	784	85.7	4681	
Secondary (10–12 years)	30.1	4437	13.2	585	86.8	3852	
College/university (13 years or more)	32.9	4862	13.8	673	86.2	4189	
Ethnicity							<0.001
Sami	23.9	3946	25.7	1014	74.3	2932	
Sami affiliation	11.4	1885	15.6	294	84.4	1591	
Non-Sami	64.6	10,649	9.1	964	90.9	9685	
Area of living							<0.001
Region 1	9.4	1548	31.1	481	68.9	1067	
Region 2	16.4	2719	11.3	306	88.7	2413	
Region 3	53.2	8809	13.3	1170	86.7	7639	
Region 4	21.0	3468	9.2	319	90.8	3149	
Church affiliation							<0.001
Church of Norway	83.7	11,121	12.8	1423	87.2	9698	
The Laestadian church	6.0	795	34.3	273	65.7	522	
Other church /religion	2.3	312	15.7	49	84.3	263	
Not a member of any church /religion	8.0	1060	8.5	90	91.5	970	

*Pearson’s chi-square test; ‘1000; ** Due to different number of respondents to the three questionnaires used in the study (Q1 = 16,544, Q2 = 15,730 and Q3 = 13,547) and missing responses to some of the questions, the number of respondents in single questions does not always add up to *n* = 16,544

### Use of traditional medicine

**Fig. 3:**
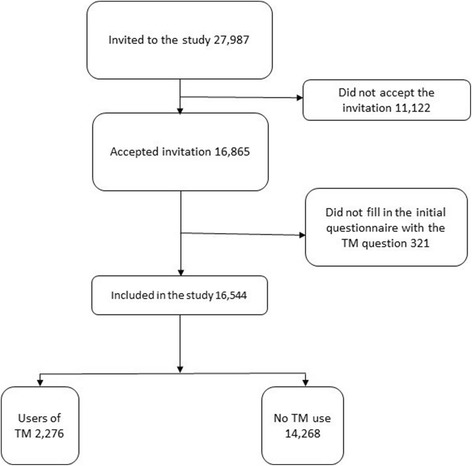
Flow chart of the included participants

From the sample of 16,544 participants included in the analyses, 5419 (32.8%) reported ever use of CAM. Of these 2276 (13.8%) reported to have visited a traditional healer (*guvllar, reader, blower, and hands on healer*) (Fig. 3). In comparison, 766 (4.6%) reported to have seen a modern healer, 2467 (14.9%) had seen an acupuncturist, and 1991 (12%) had seen other CAM providers (reflexologist/homeopath/kinesiologist, etc). Only 828 participants had used both TM and other forms of CAM. Of these, 299 had combined TM with modern healing, 508 had combined TM with acupuncture and 506 had combined TM with reflexology/homeopathy/kinesiology, etc.

#### Church affiliation

The most outstanding characteristic of the TM users was affiliation to the Laestadian church, where 34.3% (*n* = 273) of the affiliates reported to have used TM compared to 8.5–15.7% of the affiliates of other churches/religions (Table 1).

#### Area of living

Also living in region 1 (inner Finnmark) was a strong predictor for TM use, reported to be used by 31.1% (*n* = 481) of the participants from this area where 9.1% of the studied population lived. In comparison, 13.3% (*n* = 1170) of the participants living in region 3 (costal Northern Troms and Finnmark), 11.3% (*n* = 306) of the participants living in region 2 (costal Finnmark) and 9.2% (*n* = 319) of the participants living in region 4 (Nordland and Southern Troms) reported to have used TM (Table 1). Participants with inland residence were more likely to have used TM than participants with coastal residents (18.2%, *n* = 670 and 12.5%, *n* = 1606, respectively).

#### Ethnicity

Sami participants were more likely to have used TM (25.7%, *n* = 1014) compared to participants with a Sami affiliation (15.6%, *n* = 294) and non-Sami participants (9.1%, *n* = 964) (Table 1).

When Sami participants were compared to non-Sami participants, we found similar associations for TM use regarding age, gender, self-reported health and income. Differences were, however, found regarding education as more Sami than non-Sami users of TM had university education (38.4% vs. 29.7%. *p* < 0.001) (Table 1).

#### Gender, age and income

Women were slightly more likely to use TM compared to men (15.9 and 11.5%, respectively, *p* < 0.001), and the TM users were slightly younger than the non-users of TM (mean age 52.3 versus 54.3 years, *p* < 0.001). TM users also had lower income (*p* < 0.001) than the non-users of TM. We found no significant differences between TM users and non-TM users in general concerning years of education and whether the participants were living with a spouse/partner or not (Table 1).

#### Health

As shown in Table 2, most of the participants reported good health (68%, *n* = 10,565) despite the fact that many of the participants also reported health complaints. The most commonly reported complaints were pain and stiffness in muscles or joints reported by 42.7% (*n* = 6392) of the participants, followed by insomnia (35.3%, *n* = 4612) and daily cough (16%, *n* = 2134) (Table 2, left column).

**Table 2:** Health related issues in the total sample and among users and non-users of TM

	Prevalence in the total sample		Proportion using TM		Proportion not using TM		*p*-value*
	%	n**	%	n	%	n	
Current state of health							<0.001
Poor	32.0	4983	17.5	874	82.5	4109	
Good	68.0	10,565	11.7	1233	88.3	9332	
Physiological problems							
Daily cough	16.0	2134	15.0	320	85.0	1814	0.059
Heart attack	4.1	614	13.5	83	86.5	531	0.925
Angina pectoris	6.7	1006	17.4	175	82.6	831	<0.001
Cerebral stroke/brain haemorrhage	2.5	369	14.6	54	85.4	315	0.534
Multiple sclerosis	0.3	38	26.3	10	73.7	28	0.021
Fracture	11.5	1764	12.4	219	87.6	1545	0.104
Asthma	10.7	1625	18.1	294	81.9	1331	<0.001
Chronic bronchitis	4.6	694	16.4	114	83.6	580	0.052
Diabetes	4.3	654	14.1	92	85.9	562	0.037
Fibromyalgia/chronic pain syndrome	9.7	1432	20.5	294	79.5	1138	<0.001
Ulcerous colitis	1.2	175	22.3	39	77.7	136	0.001
Pain/stiffness in muscles or joints for at least three months last year	42.7	6392	15.7	1006	84.3	5386	<0.001
Psychological problems	11.7	1741	20.7	361	79.3	1380	<0.001
Sad/depressed	12.9	1699	20.3	349	79.7	1368	<0.001
Insomnia	35.3	4612	15.9	733	84.1	3879	<0.001
Unable to cope with difficulties last 14 days							<0.001
No	78.2	10,298	12.3	1262	87.7	9036	
Sometimes	19.1	2516	18.5	466	81.5	2050	
Often	1.9	249	23.7	59	76.3	190	
Almost all the time	0.8	108	26.9	29	73.1	79	
Do you sometimes feel lonely?							<0.001
No	61.1	8104	12.3	995	87.7	7109	
Sometimes	35.3	4679	15.5	726	84.5	3953	
Often	3.6	480	21.7	104	78.3	376	

* Pearson’s chi-square test ** Due to different number of respondents to the three questionnaires used in the study (Q1 = 16,544, Q2 = 15,730 and Q3 = 13,547) and missing responses to some of the questions, the number of respondents in single questions does not always add up to *n* = 16,544

More participants with poor than good health reported to have used TM (17.5%, *n* = 874 and 11.7%, *n* = 1233, respectively, *p* < 0.001) (Table 2). Among the TM users, the health problem that was most frequently reported was pain and stiffness in muscles or joints (*n* = 1006), followed by loneliness (sometimes or often) (*n* = 830) and insomnia (*n* = 733) (Table 2) representing 44%, 36% and 32% of the TM users.

Participants with multiple sclerosis reported the highest use of TM (26.3%, n = 10), followed by participants with ulcerous colitis (22.3%, *n* = 39), loneliness (often) (21.7%, *n* = 104), and psychological problems (20.7%, *n* = 361) (Table 2).

## Discussion

### Main findings

Of the 16,544 people included in the study, 2276 (13.8%) reported to have used TM at some point in their lives. Only 828 (36.4%) of these had also used other forms of CAM. The typical user of TM is a Sami woman affiliated to the Laestadian church with rather low income living in the inland of Northern Troms or Finnmark.

### Prevalence of TM

Comparisons of TM use between different studies are challenging, as questions concerning TM use, as well as the setting in which the information is collected, may differ. Previous studies of TM use among Sami and Non-Sami participants in Norway have a rather low number of participants and many include specific patient groups rather than unselected populations. To the best of our knowledge in 2017, this is the first population-based study to investigate the prevalence of TM use among Sami and Non-Sami adults in a large geographical area.

The finding of 13.8% use of TM is somewhat lower than what was found in smaller quantitative studies (34–50%) [16, 17] and qualitative studies [27–29] conducted in Northern Norway. These studies are, however, not directly comparable to our study, due to smaller number of participants, different populations and study methods. Our study is a large, unselected cohort study not limited to people with health concerns or experience with TM. In addition, as use of TM is not commonly spoken of [30, 31] use of TM might have been concealed when filling in the questionnaire. Also the fact that the TM provider often is contacted by people in the patients network [27] and not by the patients themselves, might have led to an under-report of such use due to recall bias. The patients might have forgotten that a TM provider has been contacted on their behalf, in particular in cases where the TM care was conducted as distant healing.

Our finding of 25.7% use of a TM provider among the Sami participants is somewhat lower than previous findings among Sami patients and Alaska Natives where 46–68% reported to have seen a traditional healer [17, 32]. The lower use found in our study may be due to the fact that the participants were recruited outside a health care setting among mostly healthy individuals, while the compared studies included a more selective study group.

The finding of TM use in 9.1% of the Non-Sami participants is somewhat lower than Sørlie et al. found where 37.8% of the Norwegian patients treated in a psychiatric hospital reported use of TM [17]. This is expected due to the fact that the participants in Sørlie’s study not only had a health concern, but also a psychiatric health concern that both in previous studies [28, 29] and in our study is found to be a strong predictor for TM use. The finding of 9% use of a TM provider in the Non-Sami participants is, however, in line with the use of a complementary and alternative medicine (CAM) provider in general found in two large cohort studies in mid- and Northern Norway in the same period [33, 34]. This is despite the fact that these studies included all kinds of CAM providers and not only the use of TM providers. The reason for this might be that those studies reported CAM use within the last year only, while the present study reported ever use of TM.

The highest use of TM was found among participants suffering from multiple sclerosis (MS), ulcerous colitis and psychological problems. These are all health challenges with limited treatment options within public health care, leading to a search for additional health approaches. For psychological problems, Kiil et al. found that Sami patients found the explanation and treatment of psychological disorders among TM providers more trustworthy and less stigmatizing than the treatment offered within the public health care, and more in line with their own cultural explanation model for such disease [28, 29].

### Associations for TM use

The strong association between TM use and cultural affiliation as a Sami is in accordance with findings of TM use in Native Americans [35]. As TM is considered tacit knowledge, rarely revealed to people perceived as outsiders [31, 36], the tradition might have been better kept within the Sami communities than in the Non-Sami [30, 31]. The reason for keeping the healing tradition hidden may partly be due to the witch process following the imposition of the Christianity in Norway. This led to a more hidden practice of TM as traditional healers were accused of witchery. Since the Sami people had a language not understandable for Norwegians and lived in communities often separated from Norwegian communities, the TM knowledge might have been better kept among the Sami than the Norwegian population.

One of these communities is the Laestadian church, established by the Sami priest Lars Levi Laestadius. In this church community, the sermons were conducted in Sami and the Sami culture was valued, making a safe space to continue their healing traditions. Today, the most frequently used TM in this church community is religious healing, conducted by gifted people who read bible verses over the persons illness [30]. This is in line with religious traditions across the world displaying beliefs in healing through prayer [37].

The rather high number of Non-Sami using TM in areas with Norwegian and Sami population might be due to participation in churches community where TM is actively used, and a respect for the traditional healers and their knowledge. The lack of medical doctors in the studied areas up to recent times, might have led to continued use of TM [38]. As the TM normally is offered for free or exchanged with small gifts [36], their service has been available also for people with limited financially resources. This might be one of the reasons why TM is still widely used, in particular among the participants with low income. In addition, some of the participants categorized as Non-Sami in our study, may have Sami origin. The harsh official policy to assimilate the Sami into the Norwegian culture and abandoning their Sami language was effective. Today, many people of Sami origin regard themselves as Norwegians, however, Sami culture and practices might still be present.

The high use of TM in region 1, the inland of Finnmark County, might be due to the almost exclusive Sami population and the strong tradition of reindeer herding in this area. Reindeer herding is a lifestyle including the whole family. The families have traditionally lived a nomadic life, following their reindeers. On the tundra, the absence of medical doctors was total and people needed to rely on their own knowledge when illness and injuries occurred. As the ability to heal is often inherited from older family members to younger, the families ensure that the knowledge is available when needed. The lower use of TM found in this study compared to what was found by Efskind et al. 30 years earlier [16], suggest a decrease in the TM use, despite the factors mentioned. TM practitioners express worries for the future as many now finds it more difficult than before to find family members to take over the role as a TM provider [39].

The finding of no differences between TM users and non-users of TM concerning years of education is in line with findings in recent studies for the use of CAM in general [40]. For TM in particular, this finding might suggest that the use of TM is strongly connected to the person’s tradition and identity, and not influenced by external circumstances such as formal education.

### Strengths and limitations

The main strength of this study is the large sample size (*n* = 16,544), the high response rate (60%), and the unselected sample where all residents aged 30 and aged 36–78/79 years in the selected regions were invited. As large parts of the traditional Sami settlement regions were included, the findings of use of TM among Sami people could be regarded representative of the Sami population living in Norway. However, the number of TM users with non-Sami background does not necessarily reflect the TM use among this group in general, since only rural parts of northern and mid-Norway were included. The selected municipalities cover only about 1 % of the total population of Norway.

The cross-sectional design of the study makes it difficult to suggest causal relationships between TM use and other factors studied. We can therefore not draw the conclusion that the TM reported was used for the health complaints described by the TM users. The TM might have been used before the complaint appeared, as the use of TM is continuous and not limited to a certain time frame. Further, as *traditional healer* was not defined apart from the examples of such given in the parenthesis, the participants might have varied in their understanding of how to understand the term traditional healer. Another limitation of this study is that it was conducted in 2003–2004 and therefore not necessarily reflects the TM use today. This is, however, so far the only population based study in Norway collecting data on the use of TM and will therefore be important for future studies due to the possibility to investigate trends in TM use in Northern Norway.

## Conclusions

Our study suggests that TM is widely used in Northern Norway and that people with a Sami background use more TM than Non-Sami living in the same areas. Further studies are necessary to examine the development of TM use in Norway and the use of TM should be studied also in areas with mainly Norwegian inhabitants. There is also a lack of studies describing prevalence and associations for TM use among the Sami in Sweden, Finland and Russia.

## Abbreviations

CAM: Complementary and Alternative Medicine
SAMINOR: Health and Living Conditions in Regions with Sami and Norwegian Populations
TM: Traditional medicine
